# Cervical cancer screening and treatment costing in Senegal

**DOI:** 10.11604/pamj.2024.47.151.41485

**Published:** 2024-04-02

**Authors:** Abdou Diop, Mercy Mvundura, Yacine Dieng, Malick Anne, Elisabeth Vodicka

**Affiliations:** 1 Center for Vaccine Innovation and Access, PATH, Dakar, Senegal,; 2 Product and Market Advancement, PATH, Seattle, USA,; 3 Cancer Focal Point, Non-Communicable Disease Division, Ministry of Health and Social Action, Dakar, Senegal,; 4 Non-Communicable Disease Division, Ministry of Health and Social Action, Dakar, Senegal,; 5 Center for Vaccine Innovation and Access, PATH, Seattle, USA

**Keywords:** Cervical cancer, screening, treatment, costs

## Abstract

**Introduction:**

in Senegal, cervical cancer is the leading cause of cancers among women. This study estimated the costs associated with cervical cancer screening and treatment for precancerous lesions from the health system perspective.

**Methods:**

we estimated costs for screening, diagnostics, and treatment. We conducted a cross-sectional study in seven regions with primary data collected from 50 health facilities. Data collection included structured questionnaires, with secondary data from the Ministry of Health and other sources. A mixed-methods approach combined ingredients-based costing and financial expenditures to estimate direct medical and non-medical costs. All costs are reported in 2019 USD.

**Results:**

average costs were $3.71 for visual inspection with acetic acid, $16.49 for Pap smear, and $46.65 for human papillomavirus deoxyribonucleic acid (HPV DNA) testing. Screening cost drivers were clinical exam supplies and clinical equipment for visual inspection with acetic acid, offsite processing of specimens for Pap smear, and lab equipment costs for HPV DNA procedure. The average cost of diagnosis via colposcopy alone was $25.73, and colposcopy with biopsy/endocervical curettage was $74.96. The average cost of treatment followed by one visit for pre-cancerous lesions was $195.24 for loop electrosurgical excision, $47.35 for cryotherapy, and $32.35 for thermal ablation. Clinical equipment and lab costs were the largest contributors to colposcopy and endocervical curettage/biopsy expenses. Clinical equipment made up the largest portion of cryotherapy, loop electrosurgical excision, and thermoablation costs.

**Conclusion:**

this study is the first to estimate the costs of HPV screening and treatment in Senegal, which can be used to inform decision-making on cervical cancer investments.

## Introduction

Human papillomavirus (HPV) is a globally pervasive virus that can lead to the development of cervical cancer. While efforts are underway to advance strategies toward the elimination of cervical cancer, it remains a significant health issue, particularly for low- and middle-income countries (LMICs) where nearly 90% of the disease burden occurs [1]. In 2020, the International Agency for Research on Cancer estimated approximately 604,000 new cases of cervical cancer worldwide with an age-standardized mortality rate of 7.3 per 100,000 [2]. Regionally, sub-Saharan Africa experiences the highest cervical cancer incidence and mortality [2]. In Senegal, cervical cancer is the leading cause of cancer in women, with an estimated age-standardized incidence rate of 36.3 per 100,000 women and an age-standardized death rate of 26.0 per 100,000 women per year in 2020 [3].

Fortunately, cervical cancer is preventable, diagnosable, and treatable. Safe and efficacious vaccines can provide primary prevention by protecting against HPV types that are responsible for over 70% of cervical cancers and precancerous cervical lesions. Early detection of disease can provide secondary prevention through screening and treatment of precancerous lesions to halt disease progression and reduce cancer-related morbidity and mortality. For most sub-Saharan African countries, however, implementation and scale-up of national HPV vaccination programs and screening and treatment programs remain challenging [4]. In Senegal, the HPV vaccine was introduced in 2018 for girls 9 to 13 years old, yet fewer than half of eligible girls received the vaccine in 2020 (45% and 31% for first and second doses, respectively) [3]. Low vaccination rates amplify the importance of robust national secondary prevention programs to screen women who are sexually active and at risk for developing cervical cancer but either are not eligible for the vaccine due to age or are missed during HPV vaccination program efforts.

While only 10% of women have ever been screened for cervical cancer in Senegal [3]. efforts are underway to strengthen and expand national screening and treatment programming and policy to increase capacity for potentially life-saving early detection and treatment services [5]. For example, as of 2019, over 1,000 health workers had been trained in using visual inspection with acetic acid (VIA), followed by cryotherapy as a screen-and-treat strategy. Human papillomavirus DNA testing is available at Hôpital Aristide Le Dantec reference hospital and some other tertiary care centers. Additional efforts have been underway to incorporate thermal ablation as an alternative treatment to cryotherapy in public healthcare facilities [6]. Investments by international non-governmental organizations and other institutions working locally supplement public screening programs to support the implementation of cervical cancer screening and treatment. As the Senegal Ministry of Health (MOH) and Social Action consider scale-up of services, understanding the financial and economic costs of potential programmatic and product choices can ensure that a national screening and treatment program is financially sustainable and can support value-based decision-making for future investments in cervical cancer prevention.

Recent studies have been conducted to evaluate the costs of national HPV vaccine introduction [7]. as well as the costs of routine HPV vaccination and the operational context of HPV vaccination in Senegal (results not yet published). To our knowledge, no local data are available on the costs of screening and treatment. As such, this study estimates the financial and economic costs associated with cervical cancer screening and treatment for precancerous lesions in a sub-sample of primary, secondary, and tertiary care settings from the health system perspective.

## Methods

We first conducted a high-level mapping exercise based on informal stakeholder interviews and a literature review to identify methods currently used for screening and treatment of precancerous lesions at primary, secondary, and tertiary public health facilities. This entailed informal interviews with key stakeholders such as the Cancer Focal Point and other staff from the Noncommunicable Disease Division at the MOH and reviewing the published and grey literature on cervical cancer programming in Senegal. Next, we used structured costing questionnaires to interview providers on resources used for screening and treatment of cervical cancer for each of the methods currently in use, as identified by the mapping exercise. Screening methods included VIA, Pap smear, and HPV DNA testing; diagnostic methods included colposcopy with and without biopsy and endocervical curettage (ECC); and treatment for precancerous lesions included cryotherapy, loop electrosurgical excision (LEEP), and thermal ablation. Cross-sectional resource use data were collected at the national, regional, district, and health facility levels. We applied a mixed methods approach combining ingredients-based costing and financial expenditure records review to estimate the costs per person screened, diagnosed, or treated, from the health system perspective.

**Study setting:** facilities were selected through a combination of purposive and random sampling to capture the variation in resource utilization and subsequent costs for service delivery across geographic levels (e.g., region and district) and facility type (e.g., primary, secondary, tertiary). Of the 14 regions in Senegal, we purposively selected 7 to encompass variation in terms of geography, cervical cancer screening uptake, and screening and treatment strategies being deployed. The regions included were Dakar, Thies, Saint Louis, Diourbel, Tambacounda, Ziguinchor, and Fatick. Of these, two to three health facilities within those regions were randomly selected to be included in the study. We also purposively included four facilities where HPV DNA testing pilot projects are ongoing (Dakar Centre, Guediewaye, Fatick, and Mbour) to allow for the estimation of screening with HPV DNA testing, which is not yet integrated into a standard of care in Senegal. Data provided by the Noncommunicable Disease Division at the MOH during the mapping exercise indicated that 69 health facilities and hospitals (national, regional, and district level) are currently performing different kinds of screening and treatment of cervical cancer within the study regions. Of those, 51 facilities and hospitals were included in the study from across 7 regions and 21 districts (Annex 1). In addition to health facilities, information was gathered from eight to ten laboratories that process cervical specimens, including facility-based and off-site laboratories.

**Data collection:** data were collected retrospectively in July 2022 on direct medical and non-medical costs associated with providing screening and treatment at each participating facility. Direct medical costs included clinical and laboratory personnel (i.e., staff time spent providing care, ongoing training, and supervision), supplies, and equipment. Non-direct medical costs included facility overhead. Data were collected using structured questionnaires for the staff involved in screening, diagnosis, and treatment, including (1) interviews with at least one clinical or administrative staff at health facilities to gather information on resources used for cervical cancer screening, diagnosis, and treatment; and (2) interviews with staff at contracted laboratories involved in processing cervical specimens to obtain information on resources used for specimen sampling and processing (e.g., cytotechnologists, pathologists, laboratory managers, and others). In addition, secondary data came from hospital administrators or MOH staff responsible for procuring supplies and equipment around costs of supplies and equipment used for cervical cancer screening/treatment, as well as health worker salary information. Utilization data for each procedure were obtained from health facilities, secondary data, and expert opinion. Primary data were supplemented with information from secondary data sources such as administrative reports, external references (e.g., UNICEF), literature, and reasonable assumptions when no data were available (Annex 2). Interview participants at each facility were initially referred to the study team by administrators and additional participants were identified via snowball sampling from respondents. All data were stored on a secure ODK server.

**Figure 1:**
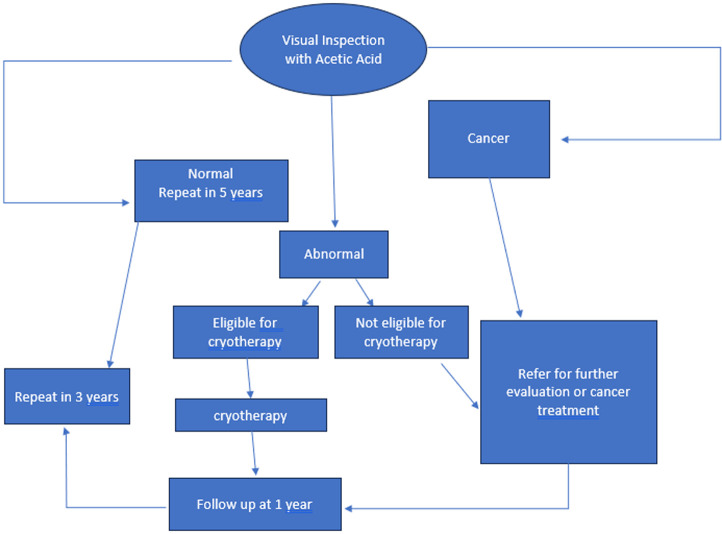
screening and treatment of women in the healthcare system in Senegal

**Data analysis:** key outcomes of the analysis were the average unit cost per person screened and the average unit cost per procedure for diagnosis and treatment. To estimate the average unit cost per screen and unit cost per procedure for diagnosis and treatment, we aggregated costs within categories. Cost categories included clinical staff, clinical exam supplies, clinical equipment, laboratory staff, laboratory supplies, laboratory equipment, and drugs. Women receiving treatment for precancerous lesions were typically asked to return for one to two follow-up visits to confirm treatment success. As a conservative assumption, we assumed that all women receiving cryotherapy, thermal ablation, and loop electrosurgical excision had one follow-up visit and applied the costs of screening at the same facility (Figure 1). For follow-up visits, we consider VIA screening for cryotherapy and thermoablation and biopsy for LEEP. Staff costs were estimated based on the average time it took to complete a screening multiplied by the average salary of typical personnel completing the procedure. In some situations where missing data existed, the lab costs were estimated through proxy measures such as costs of processing specimens at an offsite lab. Other missing data were addressed by applying data from health facilities of the same level of care with standard supplies or equipment.

Capital costs were annualized using a straight-line depreciation method and amortized for economic costs over the useful life of the product using a 3% discount rate. Opportunity costs of personnel time were estimated using facility-level salary data or Senegal´s minimum daily wage as a conservative estimate when salary data were unavailable. Any donated equipment or supplies were evaluated at market rate and depreciated or amortized, as appropriate. The useful life years used were two to ten years accordingly. We used the following formula to calculate the average direct costs for each screening or treatment modality: Total direct costs = ∑ clinical staff costs + ∑ clinical exam supplies costs + ∑ clinical equipment costs + ∑ laboratory staff costs + ∑ laboratory supplies costs + ∑ laboratory equipment costs + ∑ drugs costs + ∑ follow-up costs. All costs are reported in 2019 US dollars (USD). Conversions were made as needed using published World Bank gross domestic product (GDP) deflators for currency year and exchange rates to convert from Senegalese Francs (XOF) to USD (1 USD = 575 XOF) [8]. All data analyses were conducted in Microsoft Excel® (Redmond, Washington, United States).

**Ethical consideration:** PATH´s Research Determination Committee, Seattle, United States (REC No. 00371) and the *Comité National d´Ethique pour la Recherche en Santé (CNERS) du Sénégal*, (Approval No. 00168/MSAS/CNERS/SP) provided ethics approvals for this study. All participants underwent verbal informed consent before inclusion in the study.

## Results

Visual inspection with acetic acid was the most commonly used screening method among study facilities (n=40), followed by Pap smear (n=7) and HPV DNA testing (n=4) with average costs of $3.71 (range: $2.57-$4.88), $16.49 (range: $11.11-$24.19), and $46.65 (range: $26.86-$89.87), respectively (Table 1). Across facilities, average screening times (including lab time where relevant) were 8 minutes for VIA, 14 minutes for Pap smear, and 23 minutes for HPV DNA testing (Annex 3). The main screening cost components were clinical exam supplies (37% of total costs) and clinical equipment costs (54% of total costs) for VIA, lab supplies (26%), and costs of processing specimens at an offsite lab (38%) for Pap smear, and lab supplies (18%) and lab equipment costs (61%) for HPV DNA procedure.

**Table 1:** average aggregated direct medical screening costs by type and level of care in Senegal during initial visit (USD)

	N	Facility level	Clinical staff costs	Clinical exam supply costs	Clinical equipment costs	Lab staff costs	Lab supply costs	Lab equipment costs	Cost of processing specimens at offsite lab	Total (USD)	Min	Max
**VIA**	n=40	**Average cost**	$0.34	$1.36	$2.01	N/A	N/A	N/A	N/A	$3.71	$2.57	$4.88
**Pap smear**	n=7	**Average cost**	$0.53	$2.27	$1.57	$0.19	$4.24	$1.46	$6.22	$16.49	$11.11	$25.25
**HPV DNA test**	n=4	**Average cost**	$0.35	$5.25	$1.80	$0.19	$8.52	$28.38	$2.17	$46.65	$26.86	$89.87

VIA: visual inspection with acetic acid, human papillomavirus deoxyribonucleic acid (HPV DNA)

The average cost of diagnosis via colposcopy alone was $27.05 (range: $4.22-$36.85) and colposcopy with biopsy/ECC was $74.96 (range: $59.17-$98.56) (Table 2). The average cost of an initial treatment visit for pre-cancerous lesions plus one follow-up visit was $195.24 (range: $119.89-$251.10) for LEEP, $47.35 (range: $11.83-$77.45) for cryotherapy, and $32.35 (range: $16.97-$88.16) for thermoablation. Equipment and lab costs were the largest contributors to colposcopy + ECC/biopsy (92%); whereas clinical equipment made up the largest portion of cryotherapy, LEEP, and thermal ablation costs (96%, 94%, and 80% on average, respectively) (Table 2). Costs of treatment varied by administrative level (national, regional, and district) for the different treatment procedures (LEEP, cryotherapy, and thermoablation), though sample sizes were quite small when stratified (Table 3).

**Table 2:** average cost of diagnostic precancerous lesions by cost category and administrative level (USD)

	Diagnostics					
	Colposcopy			Colposcopy +biopsy/ECC		
	National	Regional	District	National	Regional	District
**N**	2	7	4	2	7	4
Clinical staff	0.93	0.78	0.64	1.1	1.04	0.86
Clinical supplies	0.89	0.70	1.14	3.25	4.07	3.08
Clinical equipment	10.76	28.16	34.01	11.10	31.04	34.38
Laboratory	0	0.00	0	86.98	34.78	34.78
Lab staff costs	N/A	N/A	N/A	1.12	0	0
Lab supply costs	N/A	N/A	N/A	3.65	0	0
Lab equipment costs	N/A	N/A	N/A	14.01	18	0
Costs for processing specimens at offsite lab	N/A	N/A	N/A	11.59	34.78	34.78
Medications	N/A	N/A	N/A	N/A	N/A	N/A
Follow-up	N/A	N/A	N/A	N/A	N/A	N/A
**Total costs**	12.58	29.64	34.96	45.84	70.94	73.09
**The average cost across facilities**	27.05			65.16		
**Min**	4.22			59.17		
**Max**	36.85			89.08		

**Table 3:** average costs of treatment of precancerous lesions by cost category and administrative level

Treatment of pre-cancer											
	Cryotherapy				Thermoablation				LEEP		
		National	Regional	District		National	Regional	District	National	Regional	District
**N**		1	1	2		1	4	9	1	2	3
Clinical staff		0.30	0.35	0.20		N/A	0.46	0.40	0.76	0.65	0.38
Clinical supplies		1.19	1.02	1.60		N/A	1.88	1.19	6.65	3.46	2.23
Clinical equipment		6.39	27.21	67.26		N/A	21.36	33.03	161.78	208.04	114.32
Laboratory		0.00	0.00	0.00		N/A	N/A	N/A	18.78	35.48	34.78
Lab staff costs		N/A	N/A	N/A		N/A	N/A	N/A	1.12	0.00	0.00
Lab supply costs		N/A	N/A	N/A		N/A	N/A	N/A	3.65	0.00	0.00
Lab equipment costs		N/A	N/A	N/A		N/A	N/A	N/A	14.01	0.00	0.00
Costs for processing specimens at an offsite lab		N/A	N/A	N/A		N/A	N/A	N/A	0.00	34.78	34.78
Medications		0.00	0.00	0.00		N/A	0.76	0.44	1.45	0.11	0.54
Follow-up		3.96	4.07	3.73		N/A	4.07	3.73	3.96	4.07	3.73
**Total costs**		11.84	32.65	72.80		N/A	28.53	38.79	193.38	251.10	155.98
**The average cost across facilities**	47.35				32.35				195.24		
**Min**	11.84				16.97				119,89		
**Max**	77.45				88.16				251.10		

## Discussion

This study presents the average direct medical costs of cervical cancer screening, diagnosis, and treatment of precancerous lesions from 51 health facilities in Senegal. Our findings indicate that VIA is consistently the least costly method of screening across health facilities, followed by Pap smear and HPV DNA testing. The largest cost contributors for screening were equipment and supplies (clinical and laboratory). We note the “screen and treat” strategy was mainly VIA and cryotherapy/thermoablation in these settings. Nevertheless, this model was not uniformly used since some medical staff use Pap smear as a primary screening strategy in some high-resource settings.

Several studies have evaluated the costs of care HPV and rapid diagnostic HPV tests that were introduced in several LMICs in the early 2000s. Cost estimates range from $8.52-$24.11 [9]. Other studies have evaluated the costs of molecular tests (GeneXpert, Abbott, Roche Cobas, etc.) in LMICs and found costs to be different, depending on what was included in the cost estimates.

For the treatment of precancerous lesions, we found that thermoablation was the least costly treatment for cervical precancer compared to cryotherapy and LEEP. This finding is consistent with what has been reported on costs of ablative treatment therapies within the same ranges in other LMICs [9]. To date, cryotherapy has been the most widely used ablative therapy in LMICs; however, health systems consistently report access barriers to this treatment due to gas stockouts and other inefficiencies. Newer technologies such as thermoablation present alternatives that may circumvent these barriers. As Senegal and other countries introduce or scale up the use of thermoablation, our cost estimates can be used to evaluate the potential value and financial sustainability of this investment compared to cryotherapy.

While our data came from many facilities with wide district and regional representation, it had some limitations. First, health facilities in the Demographic and Health Surveys were not routinely and accurately collecting robust data on cervical screening, diagnosis, and treatment to a large extent. Improving methods to gather routine and robust data would enhance the value of existing data, yield additional insights, and limit potential bias. Additionally, screening and treatment methods used in each location were often dependent on external non-governmental organizations (e.g. Enda Sante Cares Project, CHAI, Marie Stopes) and, therefore, may not reflect the true costs of screening and treatment programs administered by public health facilities independent of such donor engagement. In Senegal, most health facilities are still relying on VIA or Pap smears for screening [10]. Human papillomavirus DNA testing is currently only in use as part of pilot projects in select health facilities supported by donor funding. As such, our findings reflect the costs of molecular HPV DNA tests in a handful of pilot facilities and may not be reflective of the true costs of HPV DNA testing if introduced at scale. Additionally, our estimates reflect costs for multiple types of tests (GeneXpert [n=2], Roche Cobas [n=1], and Abbot [n=1]), resulting in very small sample sizes per test type (i.e., n=1-2 per test type). As such, the costs of HPV DNA testing should be interpreted with these limitations in mind. Last, the relatively small study sample size did not favor external validity and results should be viewed in this context.

Despite such limitations, our study is the first to our knowledge to estimate the costs of HPV screening and treatment in Senegal. This information can also be used to inform local decision-making on cervical cancer investments, including the ongoing costs of the program, including supplies and training, and the scalability of these interventions. These studies can also be used in cervical cancer cost-effectiveness modeling in future research. In addition, future studies would benefit from a patient-level perspective to understand household costs as well as a perspective on feasibility acceptability, and patient satisfaction. Future research should also address how different screening and treatment methodologies can enhance equitable access to services.

## Conclusion

This study reports the first known costs associated with HPV screening and treatment in Senegal. Our results are largely consistent with similar studies from other LMICs and can be used to inform decisions about investments in HPV screening and treatment in Senegal. We recommend that policymakers consider health economic evidence, including cost and cost-effectiveness, as part of a broader package of evidence for making decisions related to HPV screening and treatment in Senegal.

### What is known about this topic

- *Sub-Saharan Africa and Senegal experience some of the highest cervical cancer incidence and mortality in the world;*
- *Yet most sub-Saharan African countries have not yet optimized HPV screening and treatment programs, including cost optimization*.

### What this study adds

- *This study describes the screening and treatment methods currently available in Senegal and some of the challenges experienced;*
- *This study also reports the average cost of different screening and treatment methods in Senegal*.

## Annexes

**Annex 1**: screening and treatment activities by health facility and by region (PDF - 157 Kb)

**Annex 2**: basic assumptions (PDF - 129 Kb)

**Annex 3**: mean clinical time, capital costs, and staff costs (PDF - 117 Kb)
